# Design and Characterization of a Planar Micro-Conveyor Device Based on Cooperative Legged Piezoelectric MEMS Resonators

**DOI:** 10.3390/mi13081202

**Published:** 2022-07-28

**Authors:** Víctor Ruiz-Díez, Abdallah Ababneh, Helmut Seidel, José Luis Sánchez-Rojas

**Affiliations:** 1 Microsystems, Actuators and Sensors Lab, Universidad de Castilla-La Mancha, E-13071 Ciudad Real, Spain; joseluis.saldavero@uclm.es; 2 Electronic Engineering Department, Hijjawi Faculty for Engineering Technology, Yarmouk University, Irbid 21163, Jordan; a.ababneh@yu.edu.jo; 3 Faculty of Natural Sciences and Technology, Saarland University, 66123 Saarbrücken, Germany; seidel@lmm.uni-saarland.de

**Keywords:** standing wave, bidirectional linear motion, conveyor, nanopositioning, piezoelectric, AlN, MEMS

## Abstract

This paper reports the design, fabrication, and performance of a hybrid piezoelectric planar micro-conveyor based on Micro-Electromechanical Systems (MEMS) bridge resonators and featuring 3D-printed vertical legs. The device includes two cooperating silicon plate resonators with an area of 5 × 1 mm^2^, actuated by an integrated aluminum-nitride (AlN) piezoelectric thin film. An optimally designed array of 3D-printed projection legs was attached to the plates, to convert the standing-wave (SW) vertical vibrations into horizontal rotations or translations of the supported slider. An open-loop control strategy based on burst-type driving signals, with different numbers of sinusoidal cycles applied on each of the resonators, allowed the cooperation of the two bridges to set up prescribed trajectories of small flat objects, up to 100 mg, with positional accuracy below 100 nm and speeds up to 20 mm/s, by differential drive actuation. The effect of the leg tip and sliders’ surface finish on the conveyor performance was investigated, suggesting that further optimizations may be possible by modifying the tribological properties. Finally, the application of the micro-conveyor as a reconfigurable electronic system, driven by a preprogrammed sequence of signals, was demonstrated by delivering some surface-mount technology (SMD) parts lying on a 65 mg glass slider.

## 1. Introduction

Over the past few years, there has been an increasing demand for miniature electromechanical systems, seeking feature-rich devices that include more sensors, actuators and processors, under the same reduced size requirements [1]. The miniaturization can take these systems to the required level of integration, by downscaling the components to increase their density, and simultaneously their energy efficiency, power to weight ratio and improved response time. To help with the manufacturing of such devices, the microfactory concept could be considered a promising evolution to the traditional MEMS fabrication techniques [2].

This microfactory paradigm is closely linked to microrobotics, covering micromanipulation tools, micro-robots and micro-conveyors. One widespread requirement for all these devices is the need for precise movement, where accuracy in the order of the nanometer is often sought, together with a long travel range, large payload capacity and low energy consumption [3,4,5]. Different actuation systems, such as electrostatic, electrothermal, electromagnetic, pneumatic or piezoelectric, can be used to develop planar motion in micro-conveyors.

Electrostatic systems can be easily manufactured and scaled down with microfabrication techniques. They feature high displacement forces but at the cost of high actuation voltages while developing short strokes. In the work by Michálek et al. [6], a 1 × 1 mm^2^ unit cell micro-conveyor demonstrated precise control of the orientation of small objects submerged in fluids by inducing controlled rotations in three axes but requiring 400 V signals.

Electrothermal systems can generate large deflections with significant forces. Ebefors et al. [7] developed a bidirectional uniaxial conveyor capable of moving 3.5 g sliders at 12 mm/s with spatial resolution above 3 µm. Following the same physical principle, Ataka et al. [8] developed a closed-loop controlled 2D conveyor, consisting of 400, 1 × 1 mm^2^ unit cells. This system was able to transport and rotate 25 mg silicon slides at a maximum speed of 0.7 mm/s, with a reduced power consumption of around 136 mW per cell. As can be seen, these systems are less suitable for high-speed and low-energy-consumption applications.

Electromagnetic systems have emerged as promising planar actuators since they are contactless, relatively easy and cheap to build and exhibit large strokes with intermediate forces and response times. Arora et al. [9] developed an array of 25 × 25 mm^2^ mesh coils, capable of conveying 3.6 g objects at a maximum speed of 12 mm/s with a positional resolution of 45 µm. However, scaling down these systems can have a detrimental effect on their performance and, thus, their integrability. Recently, different approaches relying on electromagnetic actuation, combined with a stick-slip dynamic of permanent magnets, have demonstrated the conveyance of small flat non-magnetic objects [10,11]. In the work by Tisnés et al. [10], the authors presented an array of 10 × 10 mm^2^ digital electromagnetic actuators with a fine-tuned closed-loop control. The maximum conveyor speed was 2 mm/s with positional resolutions below 5 µm with 9 g sliders. Similar results were obtained by Huyan et al. [11], where a linear 6 mm path was described with deviations of less than 3% but requiring high currents and a complex design of the excitation signals.

Pneumatic systems are another viable alternative for contactless and fast conveyance. In the work of Yahiaoi et al. [12], a 9 × 9 mm^2^ monolithic conveyor based on tilted air jets demonstrated the ability to move 2 mg objects within the device plane, while a more recent work by Chen et al. [13] demonstrated speeds of 300 mm/s with positional precision of 93 µm in a closed-loop air conveyor system. Nevertheless, these actuation systems require a continuous high-pressure air source to maintain the position, and their control may be quite difficult due to the inherent complexity in developing working fluid models.

Among all the above, piezoelectric actuators have arisen as a promising alternative thanks to their cost-effectiveness, high integration, ultra-precision and immunity to electromagnetic fields [14,15,16]. Piezoelectric stick-slip systems can achieve long-range motions with sub-micrometer accuracy and high speeds. Tellers et al. [17] proposed a linear conveyor based on an array of PZT-actuated micro-hammers, capable of transporting 2 mg masses at 1 mm/s with 5 V excitation and positional errors as low as 80 µm, without speed control. In a more recent work [18], a hybrid piezoelectric micromotor, featuring 10 × 2 mm^2^ bridge resonators with attached 3D-printed legs, demonstrated linear conveyance of 21 mg sliders at a maximum speed of 35 mm/s and positional resolution below 100 nm. In this work, we take several steps further, by scaling down the system from 10 × 2 mm^2^ to 5 × 1 mm^2^, increasing the degrees of freedom (DoF) from linear movements in one axis to complete planar positioning and including an open-loop control system.

Here, we describe the design, fabrication and experimental results of a hybrid (silicon and 3D-printing technologies) planar conveyance system, suitable to translate and rotate small flat objects up to 100 mg with positional accuracy below 100 nm and speeds up to 20 mm/s. The presented micro-conveyor is based on piezoelectric MEMS resonators with attached 3D-printed legs. The plate resonators consisted of conductive silicon bridges of 5 × 1 mm^2^, actuated by an integrated aluminum-nitride (AlN) piezoelectric film sandwiched between the silicon film and the top metallic electrodes. An array of 3D-printed legs attached to the motor surface transferred the movement of the surface of the silicon plate to the slider. These legs allowed for controlled contact between the stator and slider, and the amplification of the movement caused by the resonant SW. The kinetic capabilities of the motors were characterized by the conveyance of sliders with different masses and surface roughness.

## 2. Materials and Methods

### 2.1. Device Design

**Figure 1:**
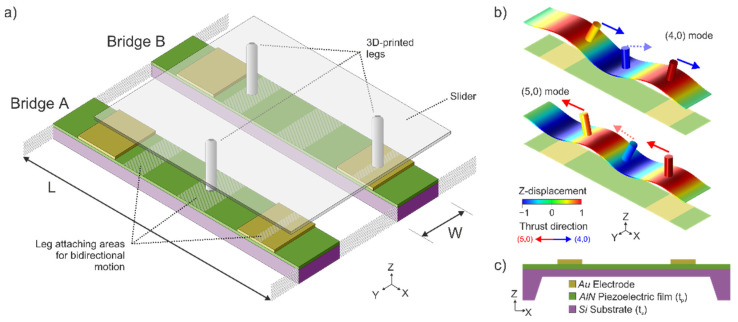
(**a**) Schematic description of the 2D MEMS conveyor. (**b**) Modal shapes for the modes involved in the motor design: (4,0) and (5,0) modes. The colored scale represents the normalized modal displacement in the *Z*-axis. Cylinders on the surface illustrate the displacement of the legs for bidirectional motion. The direction of the thrust supplied by each leg is indicated with blue or red arrows for the (4,0) or (5,0) modes, respectively. Solid and dashed arrows have 180° phase shift. (**c**) Cross-sectional schematic of the bridge resonator.

The conveyor device for planar motion consisted of a pair of cooperative linear motors based on piezoelectrically actuated bridges with a length L=5 mm and a width W=1 mm. The device layout was as follows: a 30-µm-thick p-doped (100) silicon plate served as the bottom electrode, which was covered with a 1-µm-thick aluminum-nitride (AlN) piezoelectric film. As the top electrode, two 500-nm-thick gold (Au) patches were deposited on top. Figure 1a shows the type of structure under consideration.

The motion in our planar conveyor was attained by the combination of SW generated on each of the two bridge resonators, and the appropriate design and location of several surface-attached legs [19,20]. This configuration was successfully applied recently in miniature piezoelectrically actuated robots to achieve bidirectional linear motion by means of two consecutive flexural modes [18,21]. The two bridges of our design could be considered as independent SW motors and were designed as such.

The generated SW were based on the flexural modes with four and five nodal lines along the length and no nodal lines along the width of the bridge. Using a two-index naming convention, representing the number of nodal lines along the length and the width, we will refer to these as (4,0) and (5,0) modes (Figure 1b). In order to excite optimally either of these modes, the piezoelectric patch should cover the area at which the curvature of the mode shapes has a constant sign [18,21]. Each of the resonators would have two metallic top electrodes, covering the whole width of the bridge and a length of 1.2 mm but beginning at 400 µm from each bridge anchor. This top-electrode configuration allowed for the excitation of both flexural modes on each plate, by switching the driving frequency and taking into account the parity of the modal shape for the appropriate selection of the phase difference between patches [22]. As can be seen in Figure 1b, the optimum patches cover regions of the same curvature of the (4,0) mode, so no phase difference should be applied between patches while exciting at the (4,0) mode resonant frequency. On the contrary, the optimum patches cover regions with opposite curvature of the (5,0) mode, so a 180° phase difference between both patches is required for the actuation of the (5,0) mode.

Apart from the possibility to generate two alternative harmonic SW on each plate, another key element in our conveyor design was the appropriate location and geometry of the legs that transmit the translational and rotational motion to the slider. Following the criteria of He et al. [19], the striped areas depicted in Figure 1a were considered suitable for placing these thrust-transmitting legs. As described in [18], the tip of a leg placed on these areas would describe a rectilinear trajectory, assuming that the legs were stiff enough to follow the deformation of the plate as rigid bodies. The thrust exerted by the leg to the slider would be in the positive or negative direction along the bridge length, depending on the bridge modal curvature. This can be seen in Figure 1b, where cylinders on the surface illustrate the displacement of the legs for bidirectional motion. When the resonator is excited in the (4,0) mode, the cylinders in the specified positions in Figure 1a exert thrust in the positive *x*-axis direction. On the contrary, when the resonator is excited in the (5,0) mode, the cylinders exert thrust in the negative *x*-axis direction. Therefore, to achieve bidirectional motion, two distinct flexural modes were necessary, i.e., the (4,0) mode and the (5,0) mode.

**Figure 2:**
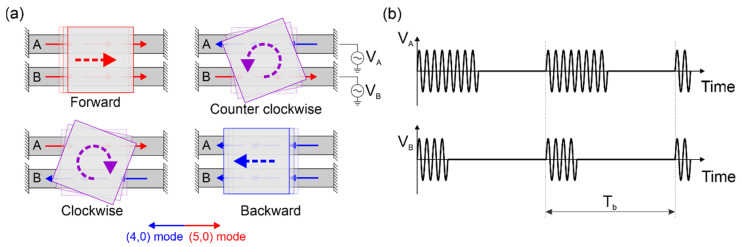
(**a**) Graphical description of the four different types of motion of the conveyor. Each bridge could be actuated either on the (4,0) or (5,0) mode for a bidirectional thrust on the supported slider. (**b**) Burst-type excitation voltage signals of period T_b_ and the same voltage amplitude applied to the bridge resonators A and B. To obtain the four types of motion, the thrust of the bridges was balanced by adjusting the number of sinusoidal cycles on A and B.

Our bi-motor conveyor design allowed for four different types of motion, as shown in Figure 2a. Each bridge could be actuated on the (4,0) or (5,0) mode, by applying sinusoidal signals at the corresponding resonant frequencies. By combining either of the modes on each of the two resonators, four distinct conveyance movements could be obtained by differential drive: forward and backward translational movement and clockwise (CW) or counterclockwise (CCW) rotation of the slider.

To implement the above four ideal types of slider conveyance, the thrust exerted by each resonator should be well balanced. Due to the asymmetries present in the structure, when the same driving signal is applied to both bridges, deviations from the ideal trajectories may be observed. Fine-tuning of the signals applied to each of the resonators was then required to balance out the unwanted deviation caused by the fabrication tolerances or non-ideal leg distribution. As can be seen in Figure 2b, our control technique relies on applying burst-type signals of period $T_{b}$, with a variable number of sinusoidal cycles at the corresponding resonance frequency of the targeted modal shape, to each one of the resonators. The ideal routes in Figure 2a were realized by adjusting the number of cycles on the burst signals for bridges A and B, independently. The voltage amplitude of the sinusoidal cycles was equal on both resonators so that the thrust could be balanced by simply tweaking the number of cycles on each plate in the active section of the burst signals.

Taking advantage of the flexibility of the 3D-printing technology employed to fabricate the legs, we also investigated the influence of their size, shape and surface finish on the motor performance. A maximum leg length was fixed so that their first resonance was higher than the driving frequencies of the robot to prevent plate–leg coupling effects. To improve the contact area between the leg and the slider, different designs were studied, such as rectangular prisms, V- and W-shaped structures and also a multiple number of cylindrical leg distributions. In addition, the effect of the surface finish of the leg tip was studied by adding different coatings.

### 2.2. Device Fabrication

**Figure 3:**
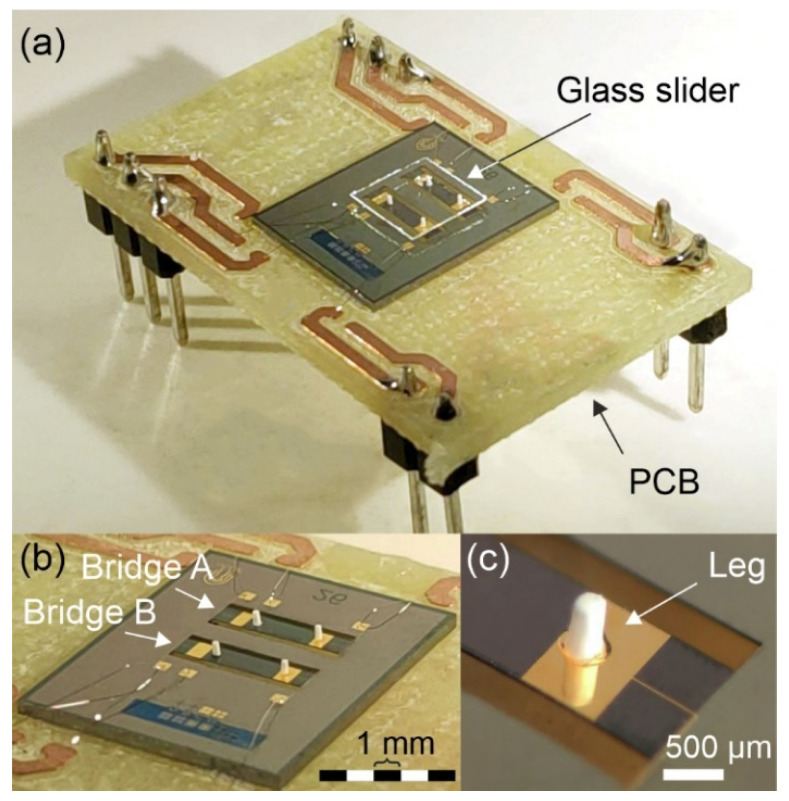
(**a**) Photograph of a dice containing the conveyor design, wire-bonded to a printed circuit board (PCB). In the experimental setup, different sliders were placed on top of the conveyor, lying on the legs. (**b**) Detailed photograph of the cooperative bridge actuators with four attached legs. (**c**) Micrograph of a 3D-printed leg, glued on the bridge surface.

Silicon micromachining techniques were used to implement the cooperative bridge actuators (Figure 3a). The fabrication of the devices included the following process steps. A highly doped p-Si wafer was thermally oxidized. The SiO_2_ on the bottom side served as an adhesion layer for a 550 nm Si_3_N_4_ film by PECVD (Plasma-enhanced chemical vapor deposition), which was used as a mask layer for the KOH etching process as part of the releasing step, defining a 30-μm-thick plate that served as the bottom electrode. The piezoelectric AlN film with a thickness of 1000 nm was deposited by reactive sputter deposition [23]. After the AlN patterns were lithographically structured, the film was etched with 85% phosphoric acid at 80 °C. The 500-nm-thick gold top electrodes were deposited via DC sputtering and etched using aqua regia at 25 °C. The resonators were released in a two-step process. In the first step, a thin membrane was created by time-controlled KOH etching from the backside. The bridges were then released using a Bosch dry etching process. The residual stress was examined for the fabricated bridges by means of an optical profiling system (NT1100 VEECO from Wyko, Tucson, AZ, United States), yielding a maximum bowing of 700 nm, which is far below the tolerances of the subsequent 3D-printing fabrication processes. Similar values were obtained for MEMS devices of the same size and fabrication procedure [24]. Dices containing two nominally equal devices were glued on a printed circuit board (PCB) and wire-bonded using a 4526 Wedge Bonder from Kulicke & Soffa (Kulicke & Soffa, Singapore) and 25-µm-thick AlSi wire, to facilitate electrical access (Figure 3b).

The resonant displacement of the surface of the silicon bridges was transferred to a slider through an array of 3D-printed legs, similar to the one depicted in Figure 3c. Various leg designs were manufactured employing a highly glass-filled resin (Rigid 10 K resin [25] from Formlabs, Somerville, MA, United States) in a Form 3 stereolithography (SLA) printer by Formlabs. This combination of resin and printer allowed a printing resolution of 200 and 50 µm, for the in-plane and out-of-plane directions, respectively. After printing, the legs were cured with ultraviolet (UV) light and heat treatment for 60 min at 70 °C, acquiring their final properties (density of $\rho =1670\;\mathrm{kg}/m^{3}$, Young’s modulus of E=10 GPa). This high-stiffness polymer-based material allowed longer legs (greater tip displacement amplitudes) with their fundamental resonance above those of the bridges, to prevent unwanted coupling effects [20]. Finally, the legs were glued to the resonator surface using a cyanoacrylate-based adhesive (Loctite, Düsseldorf, Germany) in some of the available positions depicted in Figure 1a.

### 2.3. Device Characterization

The fabricated devices were optically and electrically characterized before and after leg attachment to validate the expected performance. The electrical properties of the AlN-actuated bridges included in our conveyor device were determined by recording the impedance spectrum of the different modes of vibration. For this purpose, a 4294A Agilent impedance analyzer (Agilent Technologies, Santa Clara, CA, United States) was used. The mode identification was addressed by means of a scanning laser Doppler vibrometer (MSV 400 from Polytec GmbH, Waldbronn, Germany).

The kinetic characterization was carried out employing an optical setup consisting of a microscope camera (Throcam DCC1545M from Thorlabs, Newton, MA, United States) and a magnification tube lens with variable zoom, which allowed for different fields of view (FOV) and spatial resolutions. The FOV could be varied from 12 × 9 mm^2^ to 1 × 0.7 mm^2^, giving spatial resolutions of 4.5 µm and 300 nm, respectively. A computer-controlled signal generator (AFG 3000 series from Tektronix, Beaverton, OR, United States) was used for the required burst-type signals that generated the SW in the bridges while acquiring videos of the experiments with the camera at 30 fps.

The PCB containing the conveyor device (see Figure 3a) was placed on a levelled platform to avoid uneven movement of the slider under the microscope camera. All the subsequent experiments were performed under balanced thrust conditions, following the same compensation procedure as in [26]. The slider position and orientation versus time were finally obtained from the recorded video experiments by means of a motion tracking algorithm programmed in MATLAB (version R2022a, accessed on 15 July 2022). The figures of merit of the kinetic characterization were calculated from the resulting data.

## 3. Results

### 3.1. Motor Leg Optimization

As was previously mentioned, one of the key parameters of our SW-driven conveyor was the leg design and location. Previous works with robots based on this approach investigated the effect of the length of the legs and concluded that the longer the leg, the greater the magnification of the thrust. However, there was a limit to this length, given by the coupling of the leg’s resonance and the modal vibration of the motor. Thereby, to avoid unpredictable coupling effects, the leg length should be maintained below this limit, which in our case was around 700 µm for a diameter of 200 µm. Apart from the length of cylindrical legs, the effect of the contact area between leg and slider was investigated. For this purpose, prismatic-shaped legs (fins) and V- and W-shaped cylindrical legs (two or three tips in contact with the slider, respectively) were also designed and fabricated.

**Figure 4:**
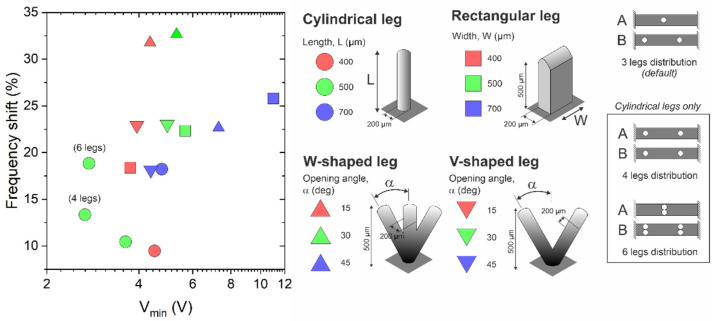
Performance of various leg shapes, sizes and distributions tested by recording the minimum voltage required to move a 10 mg silicon slider, and shift in the resonance frequency with respect to the un-legged motor. All the cases considered a 3-leg scheme similar to the one in Figure 1a, except for those labeled with (4 legs) and (6 legs).

The performance of all these leg designs was tested with square-shaped 10 mg silicon sliders, by recording the modification in the resonance frequency of the plate and the minimum excitation voltage required to move the slider one step back and forth. The mean shifts relative to the un-legged conveyor frequencies, and mean threshold voltages, are depicted in Figure 4 for each case. A lower frequency shift means less loading of the SW vibration, and hence the closer to the bottom left corner, the better the expected performance. It is expected for the larger and thicker leg structures to produce higher frequency shifts, due to the increase in the added mass and contact area with the resonator surface. On the other side, increasing the contact area with the slider (by either increasing the number of leg tips or the tip area itself) might have a beneficial effect on the minimum required voltage, associated with the higher friction force. The purpose of the variety of legs fabricated was to investigate all these effects and the implications for the actuator’s performance.

Except for the two particular cases indicated as ‘4 legs’ and ‘6 legs’, the distribution and location of the leg structures under test were as depicted in Figure 1a: one structure in the central position of bridge A, and two structures in bridge B, in the intervals that allow bidirectional motion by switching the driving mode.

As was previously mentioned, the performance of the SW-based motor would benefit from an increase in the leg length. The 500 µm leg showed a reduced minimum actuation voltage with no significant increase in the frequency shift when compared to the 400-µm-long legs. The 700-µm-long cylindrical leg showed significant coupling to the plate vibration, worsening the performance of the conveyor.

The rectangular fins and the V- and W-shaped legs were considered as alternative approaches to increase the thrust exerted by the legs, by increasing the contact area with the slider. Firstly, the fins, with a constant thickness of 200 µm, a length of 500 µm and widths from 300 to 700 µm, showed no clear improvement in the minimum actuation voltage. This could be related to the detrimental added mass effect and the increase in the glued area on the plate. In order to increase the contact area with the slider without overdamping the resonators, V- and W-shaped structures were considered. They had two and three 200-µm-diameter cylinders, respectively, with a total length of 500 µm, forming opening angles from 15° to 45°. As can be seen from Figure 4, all these structures showed frequency shifts above 20%, with no improvement in the minimum actuation voltage. This could be related to an oversized mass of the fabricated structures, especially at lower opening angles, due to the limitations of the 3D printer resolution.

Finally, two additional leg distributions were tested using the 500-µm-long, 200-µm-diameter leg. In the ‘four legs’ case, a symmetrical distribution was realized, with two legs on each resonator, located at the outer stripped areas in Figure 1a. Moreover, in the ‘six legs’ case, a pair of legs were placed on the central interval in bridge A and the two outer intervals in bridge B. As can be seen from Figure 4, the symmetrical four-leg distribution showed an improvement in the minimum actuation voltage (from 3.7 V to 2.6 V) with a small increase in the frequency shift of around 4%. Therefore, the symmetrical ‘four legs’ scheme, 500 µm in length and 200 µm in diameter, was finally chosen for the conveyor.

Once the leg distribution and shape were chosen, different surface finishing modifications were applied to the leg tip, to alter its roughness, elasticity and, ultimately, its tribological properties [27]. We tried polymer-based coatings such as polydimethylsiloxane (PDMS) and epoxy; platinum, copper and sputtered silver; and more advanced composites from mixtures of silicone-based resins with copper, aluminum or zirconia oxide powders or even graphene layers in ethanol solutions [28]. The polymer coatings showed an increase in the minimum required voltage, probably due to the softening of the tip of the leg. The rest of the coatings showed marginal improvements and further investigation might be required. Given the results from the coating experiments and for the ease of fabrication, the raw legs, as fabricated with the rigid 10 K resin without coating, were finally chosen.

### 3.2. Electrical Characterization

**Figure 5:**
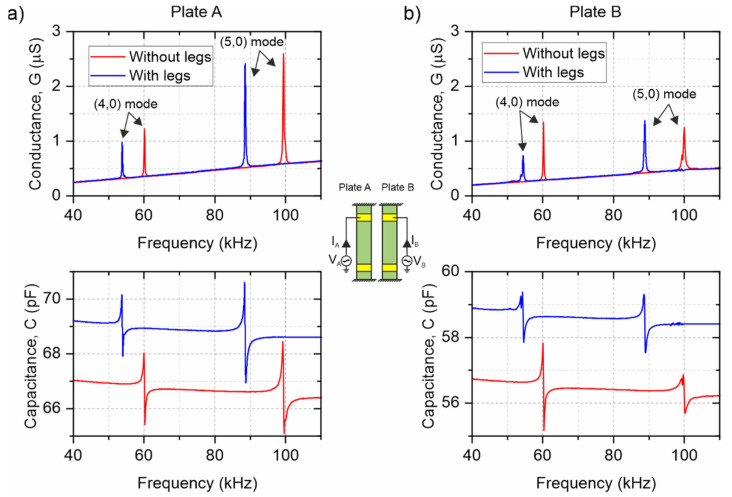
Conductance G=Re{Y(f)} and capacitance $C=\frac{Im\left\{Y\left(f\right)\right\}}{2\pi f}$ (bottom figures) from the electrical admittance spectra $\left(Y\left(f\right)=\frac{I\left(f\right)}{V\left(f\right)}\right)$ on plates A (**a**) and B (**b**) of one of the fabricated conveyors prior to (red lines) and after (blue lines) leg attachment. The main peaks were identified as the (4,0) and (5,0) modes by laser vibrometry.

Figure 5 shows the impedance spectra of one of the fabricated conveyors, before and after attaching the legs. As can be seen, two clear peaks were identified as the (4,0) and (5,0) modes. The figures of merit of these impedance peaks, such as the quality factor, the resonant frequency and the peak conductance, were deduced by fitting them to a modified Butterworth–Van Dyke equivalent electrical model circuit [29], and they are shown in Table 1. The resonant frequencies of the (4,0) and (5,0) modes were 54 and 89 kHz, respectively, while similar Q-factors around 300 and motional conductances between 1 and 3 µS were estimated. A decrease in the resonance frequencies was observed after attaching the legs, for both modes, as expected with the mass added to the structure. Furthermore, there was an increase in the quality factor that can also be associated with the increase in the effective mass of the resonator, which dominated over the augmented damping due to factors such as the alteration of the modal shape, the elastic losses in the attachment area or the interaction of the leg with the surrounding air. Similar results were obtained for all the fabricated samples with the same nominal dimensions of the plates, demonstrating the reproducibility of the process.

The fabricated devices with legs were also electrically characterized in relative vacuum (0.1 mbar) to determine the influence of the air damping on the resonator performance. As can be seen from Figure 5, a slight increase in the resonant frequency of roughly 0.5% was observed, while the Q-factor and the peak conductance almost doubled in these vacuum conditions, which may lead to better performance for the motor working with reduced air damping. Our proposed design demonstrated good performance even at atmospheric pressure, without the need for vacuum conditions, as will be seen in the following sections. Other ambient parameters, such as slight variations in the room temperature, might have a marginal influence on the resonator performance, and their effect will be neglected [30,31].

### 3.3. Kinetic Characterization

#### 3.3.1. Speed Control

**Figure 6:**
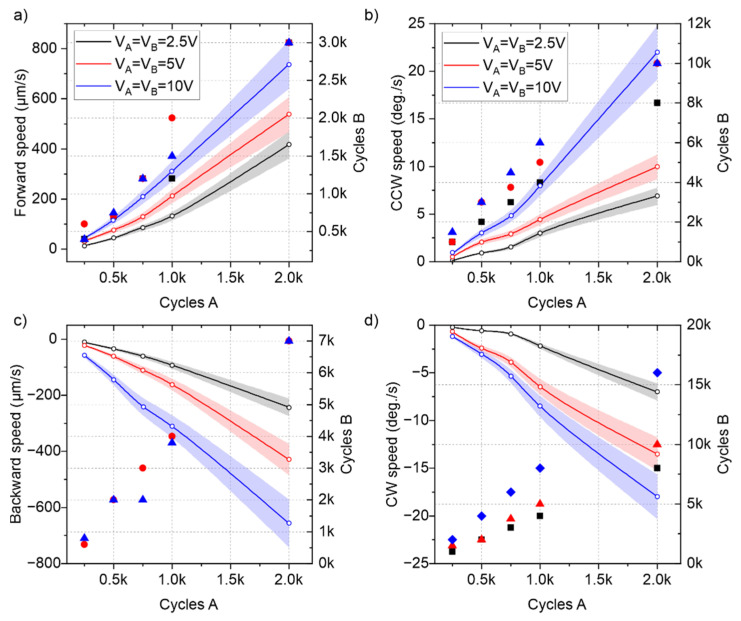
Results of translational speeds (**a**,**c**) and rotations (**b**,**d**) (solid lines and white circles related to the left vertical axes, with translucent error bands) from the experiments in the four different types of movement of Figure 2a. Each color represents a different voltage amplitude, V_A_ = V_B_, applied on both plates. The particular combinations of the number of cycles required for balanced motion are represented with markers related to the right vertical axes.

Once the bridge resonators with legs were validated individually, the cooperative actuator was tested to demonstrate the capability for trajectory control based on the driving signals. Figure 6 shows the results from the speed characterization for the four types of motion of the conveyor depicted in Figure 2a. The thrust of the plates was balanced following the same procedure as in [26]. A 10 mg silicon slider was conveyed in these experiments, performing linear displacements up to 3 mm and rotations up to 360 deg., with a controlled deviation below 1%. The burst period of the excitation signal was fixed to 1 s, and the number of periods of excitation signals applied to each plate is indicated in the right axes of Figure 6.

Figure 6a shows the mean conveyor speed for forward motion, with both plates excited at the (4,0) mode resonant frequency. The results from the balancing procedure are also indicated in Figure 6a, with markers representing the number of sinusoidal cycles driving each plate for balanced motion. A maximum speed of around 0.7 mm/s was obtained by applying 10 V and 2000 cycles on plate A and 10 V and 3000 cycles to plate B (2.26% and 3.37% duty cycle, respectively). Therefore, for balanced forward motion, plate B required 1.5 times more excitation cycles than plate A. Similar results were obtained for the backward conveyance, with plate B requiring four times more cycles than plate A but reaching a similar absolute speed. Regarding the turning capacity of the conveyor, rotational speeds as high as 20 deg./s were shown in both CW and CCW directions. On every type of motion, plate B required a higher number of sinusoidal cycles per burst period than plate A, which could be related to the lower conductance value shown in the impedance spectra (Figure 5).

**Figure 7:**
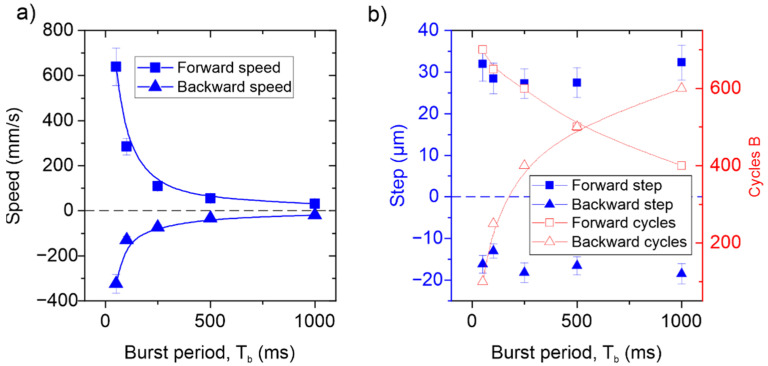
(**a**) Forward and backward translational speed as a function of the burst excitation period. (**b**) Step length and required number of excitation cycles in plate B for a compensated trajectory in the forward and backward directions.

In order to further increase the conveyor speed, apart from increasing the number of sinusoidal cycles applied on both plates, the burst signal period could be reduced. Figure 7 shows the effect of the burst period decrease in the forward and backward speed of the conveyor, for a fixed 10 V amplitude excitation and 250 sinusoidal cycles applied to plate A. As can be seen, a hyperbolic increase in speed from 30 µm/s to 600 µm/s was demonstrated by reducing the burst period from 1 s to 50 ms. Figure 7b shows the average step in the forward and backward direction per burst signal, which remains almost constant, independently of the burst period. This justifies the hyperbolic increase in the speed shown in Figure 7a. However, as the burst period was reduced, the cumulative errors in the compensation adjustment increased and the inertia of the slider became more relevant, so different ratios of the applied number of cycles to each plate were obtained. By combining these results with the ones in Figure 6, a potential translational speed as high as 20 mm/s could be achievable by decreasing the burst signal period.

#### 3.3.2. Positional Control

**Figure 8:**
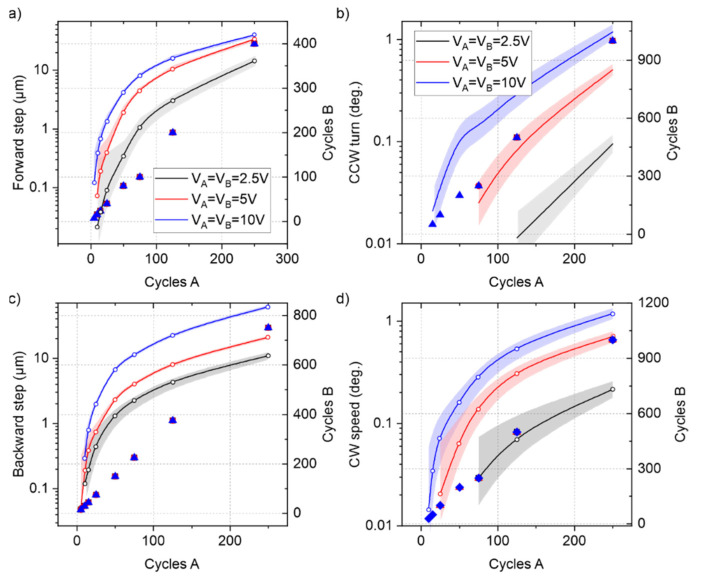
Results in terms of minimum translational steps (**a**,**c**) and turns (**b**,**d**) (solid lines and white circles related to the left vertical axes with colored error bands) from the positional experiments in the four different types of paths in Figure 2a. Each color represents a different voltage amplitude, $V_{A}=V_{B}$, applied on both plates. For a balanced trajectory, different combinations of the number of cycles, shown with markers and related to the right vertical axes, were required.

We studied the positional resolution of the conveyor by reducing the supplied energy (number of cycles) of the excitation signals in search of the smallest reproducible translation or rotation of the slider. Figure 8 shows the results from the experiments, where the same voltage was equally applied to both bridge resonators to move and rotate a 10 mg silicon slider in the smallest steps or turns possible. Here, the larger zoom was used in the optic setup, with an effective FOV of 1 × 0.7 mm^2^ and a spatial resolution as low as 300 nm. By using subpixel image processing techniques, this resolution was further decreased to 80 nm, which was considered the limit of detection (L.O.D.) of the measurement system.

The conveyor could translate the slider in steps as low as 100 nm, by decreasing the actuation voltage to 2.5 V and applying a few sinusoidal cycles. The minimum number of excitation cycles that generated motion in the slider was around 4. In addition, turns of almost 10 mdeg. were also measured. However, as the steps and turns were reduced, the errors greatly increased, due to the limitations of the optical setup. Thus, the conveyor might demonstrate an even higher positional resolution, which could be further investigated with more advanced optical techniques.

Table 2 shows a summary of the results regarding the speed and positional characteristics of the conveyor. The maximum speeds were taken from the results with a burst period $T_{b}=1\;s$ and the upper limits of the driving signals (10 V and 2000 cycles), while the minimum steps were taken from the experiments with the lower input limits of each type of motion. As can be seen, the maximum speeds at a constant burst period of $T_{b}=1\;s$ were similar for the forward and backward motions. These speeds increased as the burst period was decreased, allowing the FW speed (relying on a higher frequency resonance) to surpass the BW speed. The angular speeds were similar for the CW and CCW rotations, reaching half a turn per second with the reduction in the burst period. The minimum detectable displacements proved the positional and angular precision of the conveyor device.

#### 3.3.3. Mass Loading and Surface Roughness

**Figure 9:**
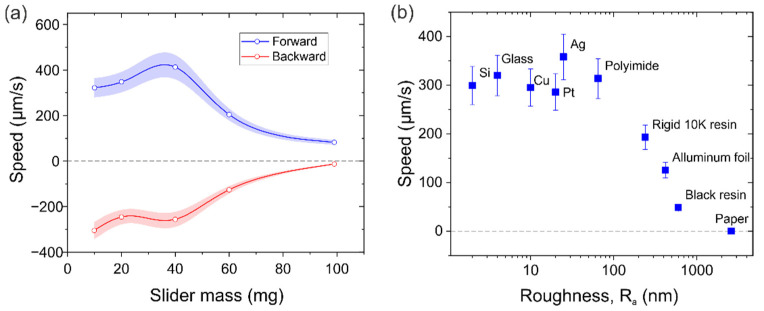
(**a**) Translational speeds in the forward and backward directions for different payloads at 10 V and 1000/1500 cycles applied to motor A/B. (**b**) Translational speed in the forward direction for sliders with the same area (5 × 5 mm^2^) and mass (10 mg), and different surface roughness.

The effect of mass loading on the performance of the conveyor was also investigated. Figure 9a shows the translational speeds for the forward and backward directions with different payloads. The same 10 V amplitude with 1000 and 1500 sinusoidal cycles per burst period was applied to bridge resonators A and B, respectively. In this case, silicon sliders with a top area of 5 × 5 mm^2^ and thickness ranging from 200 µm to 2 mm were used as payloads from 10 to 100 mg.

As can be seen from Figure 9a, the conveyor was able to move sliders of almost 100 mg, which is 25 times the combined weight of the two bridge resonators, with average forward and backward speeds of around 50 µm/s. This not only demonstrates the significant mass loading capacity of the conveyor but also supports the possible application of this design as a tethered micro-robot walker, capable of carrying its own weight. The mass loading capacity could be further increased by increasing the actuation voltage, but, due to the fragility of the thin MEMS bridges, weights above 1 g could harm the device.

It is also worth noting from Figure 9a that a maximum in the translational speed of the conveyor was reached for sliders with a mass of around 40 mg. This maximum could be attributed to a trade-off between the vibration amplitude and the frictional forces, as was already seen in other similar robot configurations [26].

Apart from the slider mass, the capabilities of the conveyor to move flat objects with different surface roughness were verified. For this purpose, sliders with the same top area of 5 × 5 mm^2^ and mass of 10 mg were fabricated in different materials, with controlled roughness. These roughness values were experimentally determined using an optical profiling system (NT1100 VEECO from Wyko, Tucson, United States). The results from the forward translational speed characterization by applying 10 V amplitude and 1000 and 1500 sinusoidal cycles with excitation signals to plates A and B, respectively, are shown in Figure 9b. As can be seen, an average speed of 0.3 mm/s was measured on all sliders with roughness below 30 nm. From this point, an increase in the slider roughness showed a decreasing conveyor speed to almost zero in the case of the paper slider. In addition, it is worth noting that the first slider to show a noticeable decrease in speed was the one made of the same material as the legs. This suggests that surfaces with a similar or greater roughness than the leg tip would exhibit an increased dynamic friction coefficient and thus a reduced conveyor speed. Further investigations on the leg tip’s interaction with the slider surface could help to overcome these roughness limitations.

#### 3.3.4. Complex Trajectory

**Figure 10:**
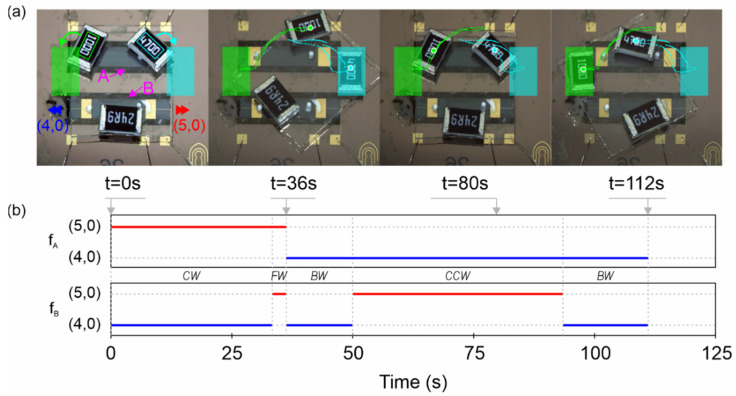
Results from the complex trajectory experiment, obtained by translating and rotating the SMD parts on the glass slider to place them on the colored areas sequentially. (**a**) Optical photograph of the conveyor, slider and SMD parts at the beginning of the sequence (t = 0 s), after locating the ‘4700’ resistor on the cyan parking area (t = 36 s), returning to the initial position (t = 80 s) and after placing the ‘1000’ resistor on the green parking area (t = 112 s). The paths described by each tracked resistor are also given, with a larger mark for each particular sequence frame. (**b**) The sequence of resonant frequencies on plate A (f_A_) and plate B (f_B_) for actuation at the (4,0) or (5,0) mode. The type of motion on each section of the sequence is also given.

Finally, we show the results from an application where the conveyor carried a 65 mg slider containing some SMD parts and moved them, replicating a reconfigurable electronic system. In this experiment, no real-time control strategy was used, but rather a sequence of preprogrammed actuation signals. The results are shown in Figure 10 and Supplementary Video S1.

As can be seen in Figure 10, the conveyor was able to place the two resistors on their corresponding parking areas, using 10 V amplitude control signals with a burst period of 1 s. The ‘4700’ resistor was conveyed to its destination (cyan rectangles in Figure 10a) in 36 s, following the dotted cyan path. Firstly, the slider was turned 45° clockwise in 33 s, following a 3 s 0.5 mm forward advance. The slider was then conveyed back to the initial position in 44 s. Next, the ‘1000’ resistor was conveyed to its parking area (green rectangles in Figure 10a) in 32 s, following the green dotted path. The slider was turned 45° counterclockwise in 15 s, following a 17 s 1 mm backward advance.

The backward motion was slower than the forward motion, as was already seen from the mass loading results in Figure 9, but each path was completed in almost the same time due to the differences in the turning speeds. The positional error was below 100 µm on the parking of both SMD resistors, which was a promising result considering that the control signals, depicted in Figure 10b, were programmed beforehand.

## 4. Conclusions

This work reports the design, fabrication and characterization of a hybrid planar conveyance system, based on piezoelectric MEMS resonators with attached 3D-printed legs. The cooperative bridge actuators consisted of conductive silicon bridges of 5 × 1 mm^2^ designed to resonate on either of two consecutive flexural modes, named (4,0) and (5,0). An array of attached legs transferred the vertical deformation of the silicon plates into horizontal rotations or translations of the slider. The effect of the leg location and geometry on the conveyor performance was investigated, resulting in a four-leg scheme of 700-µm-long, 200-µm-diameter cylinders, made of highly glass-filled resin.

Our proposed actuation technique was based on burst-type signals with a variable number of sinusoidal cycles applied on each of the bridges, allowing for the controlled planar motion, translation or rotation of different small flat objects, up to 100 mg, with positional accuracy below 100 nm and speeds up to 20 mm/s. The system demonstrated its application as a fast, low-energy conveyor for reconfigurable electronics while delivering some SMD parts contained in a 65 mg glass slider by means of a preprogrammed sequence of signals.

Finally, the effect of the leg tip and slider surface finish on the conveyor performance suggested that further optimizations may be possible by modifying the tribological properties of the surfaces in contact. In addition, the mass load capacity of the system, in combination with the low voltage amplitude signals required, might pave the way toward an autonomous walker robot with nanopositioning abilities, for future applications in ambient inspection, biomedical procedures or photonic instrumentation.

## Figures and Tables

**Table 1:** Calculated resonant frequency (f_res_), quality factor (Q) and peak conductance (ΔG), after fitting the impedance data in Figure 5 to a modified Butterworth–Van Dyke equivalent circuit model. Measurements were performed in air at atmospheric pressure and in relative vacuum (0.1 mbar).

Resonant Mode	(4,0) Mode			(5,0) Mode		
Plate	f_res_ (kHz)	Q	ΔG (µS)	f_res_ (kHz)	Q	ΔG (µS)
A (without legs) at atm	60.1	302	1.25	99.3	224	1.7
A (with legs) at atm	54	388	1.43	88.9	365	3.38
A (with legs) in vacuum	54.2	709	2.65	89.2	828	7.76
B (without legs) at atm	60.2	291	1.03	100	199	0.76
B (with legs) at atm	54	378	1.18	88.9	239	0.95
B (with legs) in vacuum	54.3	714	2.25	89.2	292	1.20

**Table 2:** Summary of the main results from the kinetic characterization of the conveyor.

Type of Motion	Maximum Speed (T_b_ = 1 s)	Maximum Estimated Speed (Minimum T_b_)	Minimum Shift
Forward	700 µm/s	20 mm/s	100 nm
Backward	650 µm/s	5 mm/s	200 nm
Counterclockwise	22 deg./s	180 deg./s	20 mdeg.
Clockwise	17 deg./s	140 deg./s	15 mdeg.

## Data Availability

Not applicable.

## Supplementary Materials

The following are available online at https://www.mdpi.com/article/10.3390/mi13081202/s1, Video S1: Preprogrammed conveyance of a 65 mg glass slider supporting several SMD parts.
